# Travel and migration associated infectious diseases morbidity in Europe, 2008

**DOI:** 10.1186/1471-2334-10-330

**Published:** 2010-11-17

**Authors:** Vanessa Field, Philippe Gautret, Patricia Schlagenhauf, Gerd-Dieter Burchard, Eric Caumes, Mogens Jensenius, Francesco Castelli, Effrossyni Gkrania-Klotsas, Leisa Weld, Rogelio Lopez-Velez, Peter de Vries, Frank von Sonnenburg, Louis Loutan, Philippe Parola

**Affiliations:** 1 InterHealth and National Travel Health Network and Centre (NaTHNaC), London, UK; 2 Service des Maladies Infectieuses et Tropicales, Hôpital Nord, AP-HM, Marseille, France; 3 University of Zürich Centre for Travel Medicine, University of Zürich, Hirschengraben 84, Zürich, Switzerland; 4 University Medical Centre Hamburg-Eppendorf, Dept. of Tropical Medicine and Bernhard-Nocht Outpatient Dept., Hamburg, Germany; 5 Service des Maladies Infectieuses et Tropicales, Hôpital Pitié-Salpétrière, Paris, France; 6 Oslo University Hospital Ullevål and University of Oslo, Oslo, Norway; 7 Institute for Infectious and Tropical Disease, University of Brescia, Brescia, Italy; 8 Addenbrooke's Hospital, Hills Road, Cambridge, UK; 9 ISTM/Geosentinel Statistician Consultant, Victoria, B.C., Canada; 10 Tropical Medicine & Clinical Parasitology. Infectious Diseases Dept., Ramón y Cajal Hospital, Madrid, Spain; 11 Division of Infectious, Tropical Medicine and AIDS, Academic Medical Centre, Amsterdam, the Netherlands; 12 Department of Infectious Diseases and Tropical Medicine, LMU University of Munich, Munich, Germany; 13 Division of International and Humanitarian Health, Geneva University Hospitals, Geneva, Switzerland

## Abstract

**Background:**

Europeans represent the majority of international travellers and clinicians encountering returned patients have an essential role in recognizing, and communicating travel-associated public health risks.

**Methods:**

To investigate the morbidity of travel associated infectious diseases in European travellers, we analysed diagnoses with demographic, clinical and travel-related predictors of disease, in 6957 ill returned travellers who presented in 2008 to EuroTravNet centres with a presumed travel associated condition.

**Results:**

Gastro-intestinal (GI) diseases accounted for 33% of illnesses, followed by febrile systemic illnesses (20%), dermatological conditions (12%) and respiratory illnesses (8%). There were 3 deaths recorded; a sepsis caused by *Escherichia coli *pyelonephritis, a dengue shock syndrome and a *Plasmodium falciparum *malaria.

GI conditions included bacterial acute diarrhea (6.9%), as well as giardiasis and amebasis (2.3%). Among febrile systemic illnesses with identified pathogens, malaria (5.4%) accounted for most cases followed by dengue (1.9%) and others including chikungunya, rickettsial diseases, leptospirosis, brucellosis, Epstein Barr virus infections, tick-borne encephalitis (TBE) and viral hepatitis. Dermatological conditions were dominated by bacterial infections, arthropod bites, cutaneous larva migrans and animal bites requiring rabies post-exposure prophylaxis and also leishmaniasis, myasis, tungiasis and one case of leprosy. Respiratory illness included 112 cases of tuberculosis including cases of multi-drug resistant or extensively drug resistant tuberculosis, 104 cases of influenza like illness, and 5 cases of Legionnaires disease. Sexually transmitted infections (STI) accounted for 0.6% of total diagnoses and included HIV infection and syphilis. A total of 165 cases of potentially vaccine preventable diseases were reported. Purpose of travel and destination specific risk factors was identified for several diagnoses such as Chagas disease in immigrant travellers from South America and *P. falciparum *malaria in immigrants from sub-Saharan Africa. Travel within Europe was also associated with health risks with distinctive profiles for Eastern and Western Europe.

**Conclusions:**

In 2008, a broad spectrum of travel associated diseases were diagnosed at EuroTravNet core sites. Diagnoses varied according to regions visited by ill travellers. The spectrum of travel associated morbidity also shows that there is a need to dispel the misconception that travel, close to home, in Europe, is without significant health risk.

## Background

In 2008, Europeans continued to represent the majority of international travellers (n = 508.7 million, 55.2%) and Europe remained the world's largest destination region (n = 489.4 million, 53%) despite suffering an overall stagnation in arrivals as compared to 2007 (+0.3%) [1]. Tourism, international business travel, immigration and travel for international aid work make up important components in the travel market. Such intense international traffic between Europe and the rest of the world, along with significant intra-European migration, results in greater vulnerability to the transmission of old, new and re-emerging infectious diseases, with travellers as a key element in disease dissemination [2].

Numerous outbreaks and case reports serve as reminders that infections can be imported and/or transmitted in Europe by visiting or returning travellers. This was illustrated by severe acute respiratory syndrome (SARS) in 2003 [3], chikungunya virus (CHIKV) in Italy 2007 [4] and most recently by the rapid spread of influenza A (H1N1) in 2009 [5]. Recognising and tracking such health threats clearly requires accurate surveillance and active collaboration allows European surveillance networks to successfully follow regional trends and detect international outbreaks of concern and provide accurate data on which evidence-based travel health policies and recommendations can be made.

EuroTravNet http://www.eurotravnet.eu, a network of clinical specialists in tropical and travel medicine was founded in 2008, to assist the European Centre for Disease Prevention & Control (ECDC) for the detection, verification, assessment and communication of communicable diseases that can be associated with travelling and specifically with tropical diseases [2]. EuroTravNet core sites also participate in surveillance and monitoring of travel-related illnesses by collecting epidemiologic data, linking diagnoses with exposure information, of returned ill travellers. Data collected using a standardised format, are entered into the international Geosentinel http://www.geosentinel.org database and analysed periodically [6]. This report describes the spectrum of infectious diseases in European travellers during 2008, grouped by specified clinical syndrome categories with focused sub-analyses of specific conditions, and emerging diseases.

## Methods

**Figure 1:**
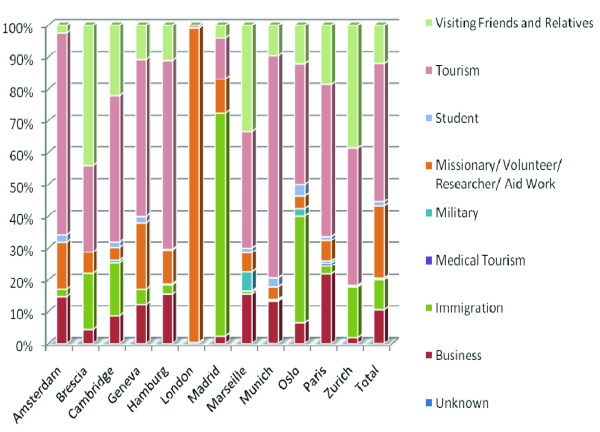
**Demographic and travel data of 6957 ill returned travellers seen in EuroTravNet core sites in 2008**.

Travellers who presented from 1^st ^January - 31^st ^December 2008 to a EuroTravNet core site (Figure 1) during or after travel were included. Data that could not be linked back to an individual patient were collected according to a standardised, anonymised questionnaire and entered by all EuroTravNet core sites into a database [7]. Reasons for travel were classified as: tourism, business, missionary/volunteer/research/aid work (MVRAs), student, military, health-care seeking, or visiting friends and relatives (VFRs) [7]. Individual countries visited were grouped into 12 regions; see figure 1. Medical data included the final diagnosis as assigned by the treating clinician, using his own diagnosis procedure and facilities, according to a standardised list of 556 possible individual diagnoses that were also categorized under 21 broad syndromes as previously described [7]. Patients were assigned as many diagnosis codes as needed. Specific diagnoses were also highlighted.

*Exposure Country *is the country specified by the consulted physician as the country where exposure most likely happened. A sub-analysis compared travellers who were likely exposed in Europe to those who were likely exposed outside of Europe. "All other travellers" includes those who were known to be exposed outside of Europe, those where no country/region was ascertainable by the physician, those who had possible exposure in more than 1 country and where the exposure countries were in different regions, or the diagnoses could not be attributed to a single travel exposure (e.g. fatigue, anaemia of an unknown aetiology). We defined *Wait Time as *the shortest time in days from the end of the last travel trip to the date of initial clinic visit, regardless of which trip resulted in potential exposure. We estimated the *total length of time spent travelling *using the sum of the durations of all the travel that had ended in the last 6 months duration, + 30 days for each trip from more than 6 months ago where they stayed at least 30 days ("Duration Sum").

Data were analyzed in SPSS v16.0^®^. In our evaluation, proportionate morbidity compares the number of cases of a specific diagnosis (or of a group of specific diagnosis within a syndrome group) to all cases of ill returned travellers seen during the same time period [6]. Differences in proportions between sub-groups ("Exposed in Europe" and "All other travellers") of ill returned travellers seen at EuroTravNet cores sites were tested using Pearson Chi-square or Fisher exact tests. Kruskal-Wallis and Mann-Whitney tests were used for quantitative variables. A significant p-value < 0.01 was chosen to adjust for the large number of statistical tests performed.

## Ethics approval

The GeoSentinel International data-collection protocol used by EuroTravNet was reviewed by the institutional review board officer at the National Center for Infectious Diseases at the Centers for Disease Control and Prevention and classified as public health surveillance and not as human-subjects research requiring submission to institutional review boards. However, where required by local regulations, the institutional review boards of each European Institutions belonging to Geosentinel/EuroTravNet reviewed and approved the GeoSentinel data-collection protocol and its use for data analyses.

## Results

**Figure 2:**
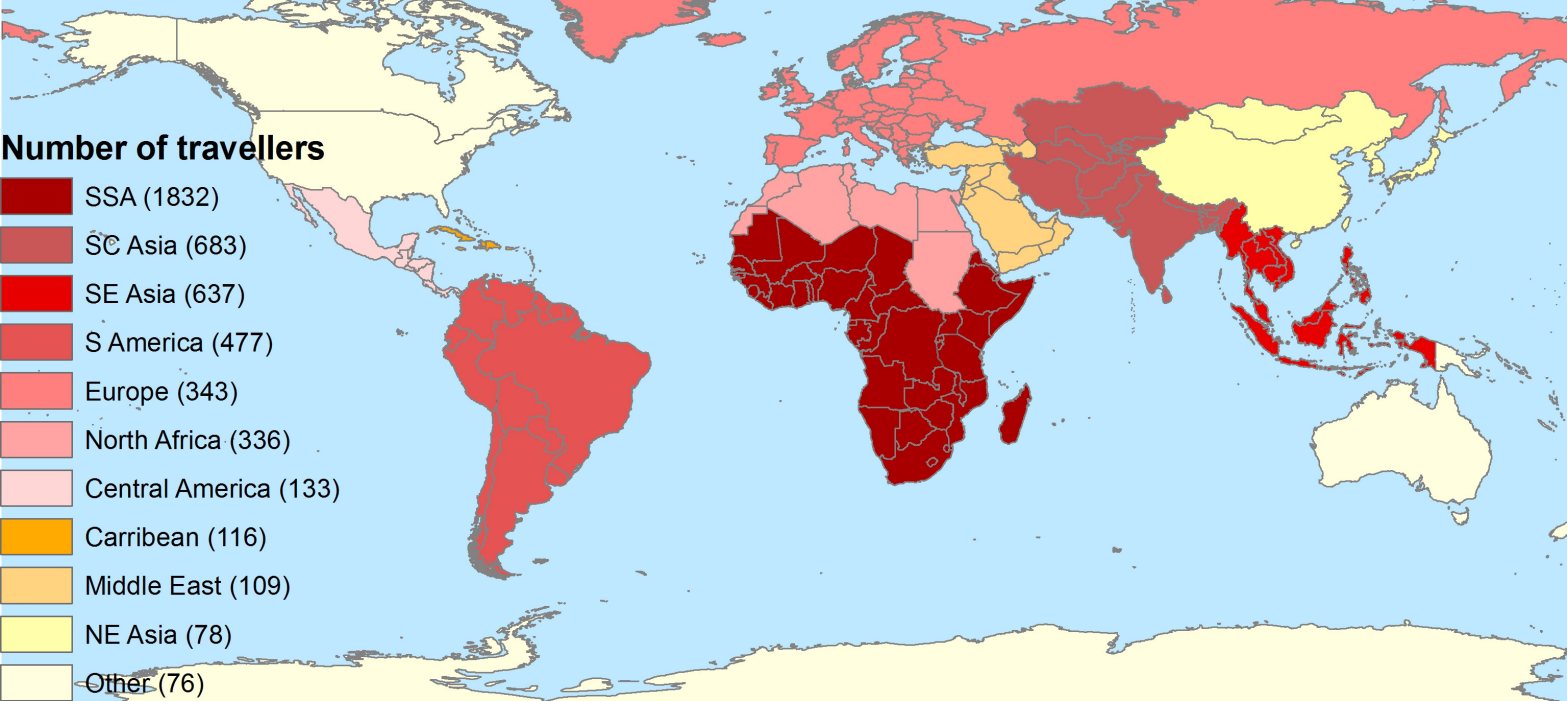
**Regions (according to GeoSentinel classification^7^) visited by 6957 ill returned travellers seen in EuroTravNet core sites in 2008**.

In 2008, EuroTravNet core sites captured data from a broad range of travellers, as shown in Figure 1. Data of 6957 ill travellers were analysed. Demographic and travel data are respectively presented in Table 1 and Figure 2.

**Table 1:** Proportion of different categories of travellers among 6957 patients seen at EuroTravNet core sites in 2008.

Patients	6957
Male	3556 (51.1%)
Female	3398 (48.9%)
Median age (min;max)	36 (0-89)
Inpatient	769 (11.1)
Outpatient	6188 (90%)
Born in Europe	5286 (76%)
Born Outside Europe	1671 (24%)
Residence in Europe	6358 (91.4%)
Residence Outside Europe	599 (8.6%)
**Travel reason**	
Tourism	3029 (43.5%)
MVRA	1569 (22.6)
VFRs	831 (11.9%)
Business	731 (10.5%)
Immigration	655 (9.4%)
Student	91 (1.3%)
Military	40 (0.6%)
Medical Tourism	10 (0.1%)
Unknown	1 (0.0%)
**Pre-travel advice**	
Yes	3160 (45.4%)
No	1554 (22.3%)
Unknown	2243 (32.2%)
**Median Duration Sum** (min-max) (days)	29 (0-14600)
**Median Wait time **(min-max) (days)	16 (0-13625)

This proportion includes 41 from Amsterdam, The Netherlands, 136 from Brescia, Italy, 126 from Cambridge, UK, , 417 from Geneva, Switzerland, 1480 from Hamburg, Germany, 1132 from London, UK, 456 from Madrid, Spain, 351 from Marseille, France, 1547 from Munich, Germany, 498 from Oslo, Norway, 548 from Paris, France, and 225 from Zurich, Switzerland. VFRs: Visiting Friends and relatives. MVRAs: missionary, volunteer, research, or aid work.

### Spectrum of diseases

Gastro-intestinal diseases accounted for 33% of illnesses, followed by febrile systemic illnesses (20%), dermatological conditions (12%) and respiratory illnesses (8%). The related morbidity of broad syndromes and selected diagnoses according to region of travel was presented in Table 2. There were three deaths in the dataset. The first case was that of a 53 year old Norwegian who died from Dengue Shock Syndrome following a three week pre-arranged tourist trip to Phuket, Thailand. The second case is that of a 57-year old aid worker who visited Liberia for three weeks. He was admitted with coma as a result of severe malaria, a week after return to Geneva, and subsequently acquired a nosocomial infection and died of septic shock. The third case was a 67 year old Norwegian who died from *E.coli *pyelonephritis and sepsis, following a two month visit to Spain; details of underlying pathology are not known.

**Table 2:** Selected syndrome groups according to travel region among 6957 patients seen at EuroTravNet core sites in 2008.

	Total N %	Caribbean	Central America	Europe	Middle East	North Africa	North East Asia	Other	South America	South Central Asia	South East Asia	Sub-Saharan Africa	No single region
**Febrile/Systemic Illness**	1378	23	21	46	16	22	17	9	70	116	160	602	276

	20%	20%	16%	14%	15%	7%	22%	12%	15%	17%	25%	33%	13%

**Acute Diarrhea**	1205	27	42	67	33	119	15	6	70	263	131	319	113

	17%	24%	32%	20%	31%	36%	19%	8%	15%	39%	21%	18%	5%

**Chronic Diarrhea**	484	9	14	11	10	53	5	4	41	71	57	85	124

	7%	8%	11%	3%	9%	16%	6%	6%	9%	11%	9%	5%	6%

**Gastrointestinal Other**	623	8	16	30	12	29	11	6	44	70	44	176	177

	9%	7%	12%	9%	11%	9%	14%	8%	9%	10%	7%	10%	8%

**Dermatologic**	851	35	34	62	14	43	9	27	83	72	140	216	116

	12%	30%	26%	18%	13%	13%	12%	37%	18%	11%	22%	12%	5%

**Respiratory**	540	8	6	72	10	15	4	8	18	65	65	119	150

	8%	7%	5%	21%	9%	5%	5%	11%	4%	10%	10%	7%	7%

**Tissue Parasites**	237	0	0	5	2	10	0	1	96	2	3	85	33

	3%	0%	0%	2%	2%	3%	0%	1%	20%	0%	1%	5%	2%

**GU and STIs**	200	6	2	16	2	7	1	0	11	17	23	44	71

	3%	5%	2%	5%	2%	2%	1%	0%	2%	3%	4%	2%	3%

**Psychologic**	175	2	0	6	3	14	5	0	7	18	5	52	63

	3%	2%	0%	2%	3%	4%	6%	0%	2%	3%	1%	3%	3%

**Injury/** **Musculoskeletal**	144	2	1	16	3	9	9	5	10	13	13	24	39

	2%	2%	1%	5%	3%	3%	12%	7%	2%	2%	2%	1%	2%

**Neurologic**	98	1	0	13	3	7	1	3	3	8	8	22	29

	1%	1%	0%	4%	3%	2%	1%	4%	1%	1%	1%	1%	1%

**Death**	3	0	0	1	0	0	0	0	0	0	1	1	0

	0%	0%	0%	0%	0%	0%	0%	0%	0%	0%	0%	0%	0%

**Chronic Disease**	319	1	1	17	1	4	4	2	61	9	13	107	99

	5%	1%	1%	5%	1%	1%	5%	3%	13%	1%	2%	6%	5%

**Nonspecific Symptoms**	283	4	6	8	2	13	3	3	38	14	19	91	82

	4%	4%	5%	2%	2%	4%	4%	4%	8%	2%	3%	5%	4%

**Other**	347	6	11	18	6	17	5	7	27	24	42	107	77

	5%	5%	8%	5%	6%	5%	6%	9%	6%	4%	7%	6%	4%

**Healthy**	1056	1	3	3	5	5	1	0	8	9	4	36	981

	15%	1%	2%	1%	5%	2%	1%	0%	2%	1%	1%	2%	45%

**Total**	6957	115	133	341	107	330	78	73	470	676	631	1804	2199

	100%	100%	100%	100%	100%	100%	100%	100%	100%	100%	100%	100%	100%

### Etiologic diagnosis

The related morbidity of selected diagnoses according to region of travel is presented in Table 3. Acute diarrhea was the most frequent gastro-intestinal concern and acquired from all traveled regions although there are regional differences. Among identified pathogens, *Shigella spp *accounted for most cases. There were 284 pathogenic intestinal protozoan infections reported including 193 *Giardia intestinalis *(the second most common identified pathogen responsible of acute diarrhea), 16 cases of cryptosporidiosis, mostly from Egypt and India, and 12 cases of cyclosporiasis (worldwide distribution). Within 156 cases of helminthic infections, 54 cases of strongyloidiasis and 10 cases of loiasis were reported. Nine cases of hepatitis E were diagnosed including 4 cases from Pakistan, 2 from India, 2 from Bangladesh, and 1 from Sudan. A total of 129 cases of schistosomiasis were reported, the majority acquired by MVRAs (48.8%; n = 63), tourists (19.4%; n = 25) and VFRs (14.0%; n = 18), in sub-Saharan Africa (SSA) (61.2%; n = 79), namely Uganda (n = 15), Tanzania (n = 8), Malawi and Ghana (n = 7) and North Africa (10.1%; n = 13), namely Egypt (n = 8) and Sudan (n = 5). The majority of schistosomiasis cases (n = 113) did not have species specific diagnoses; there were 8 cases of *Schistosoma mansoni *(mostly from SSA), and 8 cases of *S. haematobium *(mostly from SSA).

**Table 3:** Selected etiologic diagnoses according to travel region among 6957 patients seen at EuroTravNet core sites in 2008.

	Total N %	Caribbean	Central America	Europe	Middle East	North Africa	North East Asia	Other	South America	South Central Asia	South East Asia	Sub-Saharan Africa	No single region
**Acute Diarrhea**	1205	27	42	67	33	119	15	6	70	263	131	319	113

	17%	24%	32%	20%	31%	36%	19%	8%	15%	39%	21%	18%	5%

*Campylobacter*	87	0	0	4	6	6	2	0	4	25	17	16	7

	1%	0%	0%	1%	6%	2%	3%	0%	1%	4%	3%	1%	0%

*Shigella*	333	5	8	13	11	27	4	1	12	76	31	108	37

	5%	4%	6%	4%	10%	8%	5%	1%	3%	11%	5%	6%	2%

*Salmonella NT*	59	1	1	5	1	7	1	0	3	5	7	19	9

	1%	1%	1%	2%	1%	2%	1%	0%	1%	1%	1%	1%	0%

*Giardia*	193	2	5	7	4	7	0	0	13	74	15	47	19

	3%	2%	4%	2%	4%	2%	0%	0%	3%	11%	2%	3%	1%

*Amebas*	25	1	1	0	0	1	0	0	2	10	1	7	2

	0%	1%	1%	0%	0%	0%	0%	0%	0%	2%	0%	0%	0%

**Febrile**	1378	23	21	46	16	22	17	9	70	116	160	602	276

Systemic*	20%	20%	16%	14%	15%	7%	22%	12%	15%	17%	25%	33%	13%

*P. falciparum *Malaria	255	2	0	0	0	2	0	0	1	3	0	241	6

	4%	2%	0%	0%	0%	1%	0%	0%	0%	0%	0%	13%	0%

Malaria other	116	0	0	0	0	2	0	3	11	12	1	82	5

	2%	0%	0%	0%	0%	1%	0%	4%	2%	2%	0%	5%	0%

Dengue	131	6	7	0	0	1	1	0	13	21	60	9	13

	2%	5%	5%	0%	0%	0%	1%	0%	3%	3%	10%	1%	1%

Chikungunya	12	0	0	0	0	0	0	0	0	4	0	8	0

	0%	0%	0%	0%	0%	0%	0%	0%	0%	1%	0%	0%	0%

Rickettsiosis	50	0	0	6	1	0	2	0	1	1	0	36	3

	1%	0%	0%	2%	1%	0%	3%	0%	0%	0%	0%	2%	0%

Salmonellosis	20	0	0	1	0	1	0	0	0	8	2	6	2

	0%	0%	0%	0%	0%	0%	0%	0%	0%	1%	0%	0%	0%

**Dermatologic**	851	35	34	62	14	43	9	27	83	72	140	216	116

	12%	30%	26%	18%	13%	13%	12%	37%	18%	11%	22%	12%	5%

Rabies PEP	65	0	2	6	4	19	1	2	5	2	17	7	0

	1%	0%	2%	2%	4%	6%	1%	3%	1%	0%	3%	0%	0%

Arthropods' bite	177	9	5	24	3	5	2	7	18	14	43	37	10

	3%	8%	4%	7%	3%	2%	3%	10%	4%	2%	7%	2%	1%

Larva migrans	97	3	6	1	0	1	0	2	17	10	24	28	5

	1%	3%	5%	0%	0%	0%	0%	3%	4%	2%	4%	2%	0%

Bacterial infection	229	14	11	15	5	6	2	9	13	23	33	63	35

	3%	12%	8%	4%	5%	2%	3%	12%	3%	3%	5%	4%	2%

Leishmaniasis	15	0	2	1	0	3	0	0	3	1	0	0	5

	0%	0%	2%	0%	0%	1%	0%	0%	1%	0%	0%	0%	0%

Myiasis	22	0	3	0	0	0	0	0	6	0	0	12	1

	0%	0%	2%	0%	0%	0%	0%	0%	1%	0%	0%	1%	0%

**Respiratory**	540	8	6	72	10	15	4	8	18	65	65	119	150

	8%	7%	5%	21%	9%	5%	5%	11%	4%	10%	10%	7%	7%

**STIs**	42	0	0	4	0	1	0	0	3	1	6	10	17

	1%	0%	0%	1%	0%	0%	0%	0%	1%	0%	1%	1%	1%

**Schistosomiasis**	129	0	0	2	1	11	0	0	2	2	2	79	30

	2%	0%	0%	1%	1%	3%	0%	0%	0%	0%	0%	4%	1%

Cases of dengue reported by EuroTravNet core sites in Brescia, Italy and Oslo, Sweden have been included in [34]

**Figure 3:**
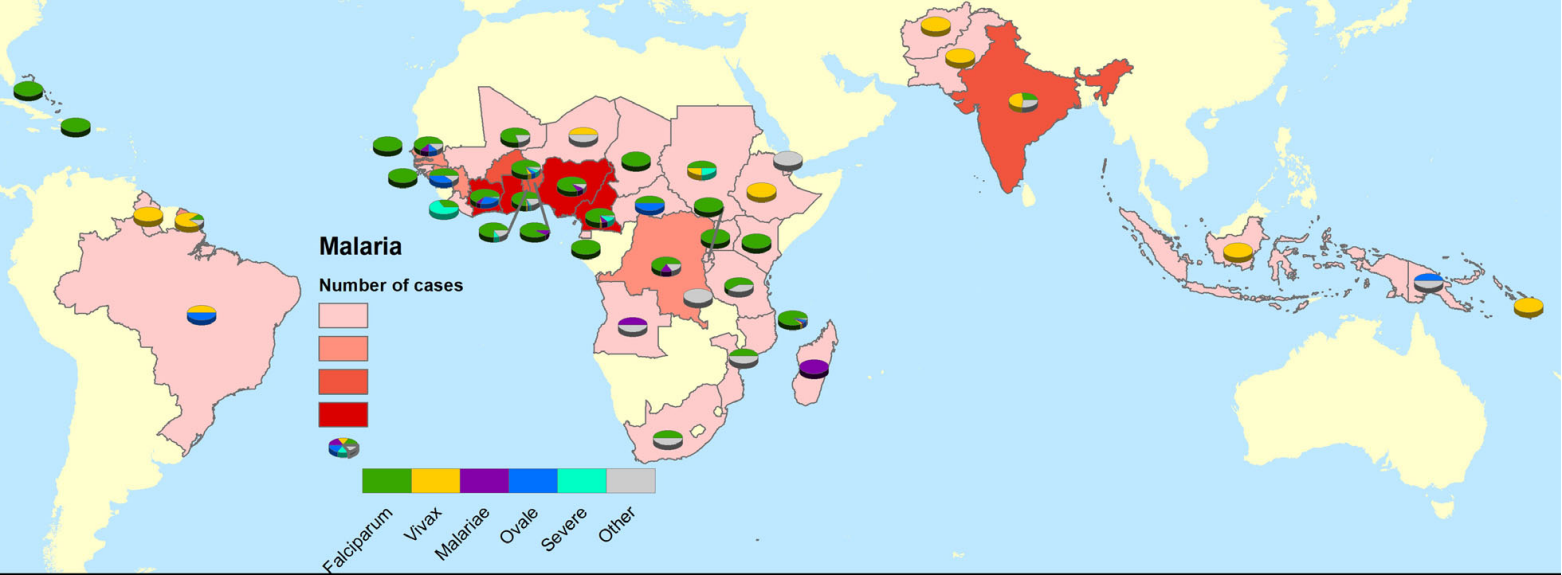
**Countries of acquisition of malaria in ill returned travellers to EuroTravNet core sites in 2008**.

Among febrile systemic illnesses with an identified pathogen, malaria accounted for most cases followed by dengue. A total of 371 cases of uncomplicated malaria were reported including 255 *Plasmodium falciparum *infections; an additional 5 cases were reported as severe complicated cerebral malaria and 7 cases as severe, complicated non-cerebral malaria. One of these cases proved fatal. The majority of *P. falciparum *(87.1%) was acquired in SSA, namely Comoros Islands, Ghana, Ivory Coast, Cameroon and Nigeria, and by VFRs (65.9%). Also, 31 cases of *P. vivax *(mostly from South Central Asia (India, Pakistan, Afghanistan), South America (French Guiana and Brazil), and SSA), 25 cases of *P. ovale *(mostly SSA (Ivory Coast, Comoros, Guinea)) and 14 cases of *P. malariae *(all from SSA) were diagnosed. The countries of acquisition of malaria in our returned travellers are shown in Figure 3.

Dengue was diagnosed in 131 patients with 1 death. The majority (45.8%) of dengue cases were acquired in South East Asia, notably Thailand (n = 32), Indonesia (n = 7), Myanmar (n = 4) and Vietnam (n = 6). 16.0% was acquired in South Central Asia, notably India (n = 14), Maldives (n = 4) and Sri Lanka (n = 2); 9.9% were acquired in South America, notably Brazil (n = 7); 6.9% of cases were acquired in SSA (Burkina Faso, Cameroon, Cote D'Ivoire, Gabon, Mali and Senegal). The majority were diagnosed in tourists (61.1%) and imported into Germany (58.8%) and France (17.6%).

A total of 12 cases of chikungunya virus (CHIKV) were acquired in Indian Ocean Islands of Reunion (n = 3), Comoros (n = 2), Sri Lanka (n = 2), Madagascar (n = 1), Maldives (n = 1), as well as from India (n = 1). Cases were also imported from Kenya (n = 1) and Senegal (n = 1). The majority were diagnosed in tourists and imported into France and Germany.

Among 50 cases of reported rickettsial diseases (74% in tourists), spotted fever group rickettsioses accounted for the majority of cases (86.0%); most of these were African tick bite fever acquired in South Africa (n = 28), Tanzania, Mozambique, Uganda, Namibia, Kenya, Ethiopia and Gambia. Mediterranean spotted fever was diagnosed in 4 cases, acquired in France (n = 2), Greece (n = 1) and Spain (n = 1). One case of scrub typhus was reported from India.

Other febrile illnesses included 7 cases of leptospirosis that were acquired in Cambodia (n = 2), Cameroon (n = 1), Central African Republic (n = 1), Costa Rica (n = 1), Indonesia (n = 1) and Reunion (n = 1), by tourists (n = 4) and business travellers (n = 3); two cases of acute brucellosis, respectively in a tourist following a trip to Thailand and in a VFR who was exposed on a return visit to his country of birth Algeria; three cases of toxoplasmosis, 20 cases of acute Epstein Barr Virus infection, and one case of histoplasmosis.

Bacterial infections accounted for most dermatological diagnoses followed by arthropod bites and cutaneous larva migrans (Table 2). Also, 80 animal bites (including dog, cat and monkey bites) were reported including 65 (81.3%) known to have been treated with rabies post-exposure prophylaxis (PEP). The majority of exposures occurred in Thailand (16.3%; n = 13), Algeria (13.8%; n = 11) and Morocco (8.8%; n = 7) and were seen in tourists (68.8%) and VFRs (22.5%).

A total of 15 cases of leishmaniasis, 12 cutaneous, 1 mucocutaneous, and 2 visceral, were reported. Cases of cutaneous leishmaniasis were acquired in North Africa (Morocco n = 2, Sudan n = 2), Central and South America (Costa Rica n = 1, Ecuador n = 1, Guatemala n = 1, Peru n = 1), South Central Asia (Afghanistan n = 1), and Western Europe (Malta n = 1); two exposures were unascertainable. The case of mucocutaneous leishmaniasis was acquired in Venezuela. One case of visceral leishmaniasis was acquired in Spain. The majority of cases, 66.7%, were acquired by tourists (n = 10) and imported into Germany (n = 9). Other dermatological diagnosis reported included 22 cases of myiasis, 13 cases of tungiasis, and one case of leprosy in a 24 year old Somalian-born immigrant to Switzerland.

Respiratory illnesses that were reported included 104 Influenza-like illnesses (ILIs), and 8 cases of influenza A and 2 cases of influenza B. Of the 68 cases with a reported country of exposure, the majority (n = 43) were exposed in SSA. There were 112 cases of *Mycobacterium tuberculosis *(TB) infections including 64 in immigrants, 32 in VFRs (mostly foreign-born), 6 tourists, 1 student, 1 MVRAs and 8 business travellers. There were 39 cases of pulmonary TB, 6 cases were of MDR or XDR TB, 5 of TB meningitis, 4 of CNS tuberculoma, 19 of disseminated/miliary TB, and 39 other cases reported as extrapulmonary TB. Also, 5 cases of Legionnaire's disease were mostly in tourists, including 2 with known exposures in Spain.

Chagas disease (*Trypanosoma cruzi*) was diagnosed in 94 patients. The majority were exposed in Bolivia (95.7%; n = 90), and imported into Spain (97.9%; n = 92) by immigrant travellers (98.9%; n = 93). Two cases were imported from Ecuador, 1 from Argentina and 1 from Paraguay. Most cases (95.7%; n = 90) were confirmed when screening patients with no specific complaints of Chagas disease. Two cases presented with musculoskeletal pain and two with gastrointestinal symptoms.

The proportionate morbidity of genito-urinary and sexual transmitted infections (STIs) was 2.9%. A total of 52 newly-diagnosed asymptomatic HIV cases were reported, mostly in immigrants (n = 32) but also 10 in tourists, 6 in VFRs, 3 in students, and 1 in a business traveller. There was a further 43 cases of asymptomatic HIV, and 32 cases of AIDS, reported mostly in immigrants and VFRs. Place of exposure was difficult to ascertain. Also, 21 cases of syphilis were reported, the majority in male (n = 16), immigrant or foreign-born VFR travellers (n = 14).

Finally, there were 166 cases of potentially vaccine preventable diseases reported including 23 cases of acute hepatitis A, 9 cases of acute hepatitis B, 2 cases of measles, 4 cases of *Haemophilus influenzae *B meningitis, 1 case of pneumococcal meningitis, 3 cases of pertussis, 1 case of tick borne encephalitis (from Estonia), 9 cases of typhoid fever, 4 cases of acute varicella, and the cases of influenza A (8 patients) and influenza B (2 patients) presented above. The majority of these illnesses were acquired by tourists and business travellers. Immigrants and VFRs travellers accounted for the majority of cases of chronic hepatitis B carriage (n = 99), diphtheria (n = 2) and TBE (n = 1). The cases of diphtheria were those of a 5 year old boy, born and raised in Norway, who travelled with his mother to her homeland to visit friends and relatives in Latvia. The boy presented with fever and ear, nose and throat symptoms, one week after their return and was diagnosed with diphtheria. His mother was positive on asymptomatic screening.

### European travel sub-set

When comparing travellers who were likely exposed in Europe to those who were likely exposed outside of Europe, significant differences (p < 0.01) were found in relation to several variables. Those exposed in Europe were more likely to have been born in Europe, currently live in Europe, be younger, not seek pre-travel advice, travel for shorter trips, be VFRs or tourists, be more likely to require inpatient care, and were less likely to be business travellers, or MVRAs. There were no significant differences between those exposed in Europe and all other travellers in relation to gender, or time to presentation. These results were not affected by excluding or including patients who came to a clinic for asymptomatic screening, usually after missionary volunteer work and were found to be healthy (n = 953).

In those Europeans who returned from a trip within Europe, 16.9% had acquired a gastro-intestinal illness (including acute and chronic unspecified diarrhea, giardiasis, *Campylobacter *infection and non-typhi salmonellosis); 14% had acquired a respiratory illness (including bacterial pneumonia, upper respiratory tract infection, acute bronchitis and atypical pneumonia); and 7.9% had a dermatological condition (including insect bite, dog bite, tick bite, skin abscess and infection).

Of those Europeans who were exposed in Western Europe (n = 283), the majority were tourists (n = 204), travelling from where they lived in Norway (n = 37), Germany (n = 34), or Switzerland (n = 32), and France (n = 22), to Spain (n = 83), Germany (n = 74) and Italy (n = 43); 27.2% (n = 77) had sought pre-travel advice. The most common diagnoses were respiratory illness (11%), gastro-intestinal illness (10.9%) and insect bites (5.7%). Of those Europeans who were exposed in Eastern Europe (n = 145), the majority was VFRs (n = 83), travelling from where they lived in Switzerland (n = 72) or Germany (n = 20), to Serbia (n = 29), Russia (n = 20), Albania (n = 18), Macedonia (n = 12) and Croatia (n = 9).

Gastro-intestinal illness was reported in 22.1% of patients who travelled to Eastern Europe, compared to 10.9% in those who travelled to Western Europe. Respiratory illness accounted for 14.5% of diagnoses from Eastern Europe compared to 11% from Western Europe. Dog bites resulting in rabies post-exposure treatment was reported in 9 cases (6%) following travel to Eastern Europe. Insect bites were reported in 8 cases following travel to Western Europe.

Additionally, in those who travelled to Western Europe as compared to Eastern Europe, more cases of potentially vaccine preventable illness (including influenza A and B, pneumococcal meningitis, *Salmonella typhi *and varicella) and acute STIs (including acute symptomatic and asymptomatic newly diagnosed HIV and chlamydia) were reported. Four cases of ricketssial disease, Mediterranean Spotted Fever, were also diagnosed. More cases of TB (including MDR or XDR, pulmonary, disseminated, meningitis, extrapulmonary and asymptomatic tuberculin PPD positive), acute hepatitis A and tick bites were reported in those who travelled to Eastern Europe as compared to Western Europe.

## Discussion

EuroTravNet captures a sentinel sample of travellers. This analysis details the complex epidemiology of travel associated morbidity in European travellers and highlights the different profiles of travel-acquired illness in different types of travellers. Furthermore, the spectrum of disease associated with travel within Europe (Eastern and Western Europe) is evaluated and risk areas and groups are identified. Each EuroTravnet site has specific characteristics, and some could be considered as sentinel sites for diseases in specific categories of travelers returning from particular countries. However, surveillance in European travelers that encompasses a wide range of sites in Europe, including some with local specificity, is crucial to determine the epidemiology of travel-associated disease, to detect alarming events, and, if required, to organize a rapid response [6]. The major strengths of our analysis are its multi-centre nature providing a large number of participants from twelve European countries, capturing all traveller types, and its focus on proportionate disease as a risk indicator. The limitations of this method of risk analysis have been recently discussed [6]. Particularly, because the denominator data (numbers of travellers) cannot be ascertained, it is not possible to calculate incidence rates. However, despite this limitation, the EuroTravNet/Geosentinel database analysis has been identified as a main source of data on the epidemiology of travel-related illness [6-10]. Because of the lack of data collected on injuries and accidents, which are frequent causes of travel associated morbidity and mortality, we focused here on infectious diseases morbidity. We selected and discussed specific causes among the main syndromes affecting European travellers, including gastro-intestinal disorders, febrile systemic illness, dermatological problems and respiratory diseases, focusing on emerging diseases or diseases of importance for public health in Europe.

During the 20th century, malaria was eradicated from the whole of the European Union (EU). The potential for the reappearance is a constant concern as potential vectors remain present. Continued surveillance remains important to update guidelines for malaria chemoprophylaxis, as malaria remains the first common specific diagnosis in febrile returned travellers. Since 2000, a slight but steady decrease in the overall incidence rates of imported malaria in Europe has been observed; however, all evaluations of imported malaria show an increasing proportion of life-threatening *P. falciparum *malaria in certain risk groups such as those visiting friends and relatives, as shown here [11,12]. Our data show VFRs returning from SSA continue to be those most at risk of *P. falciparum *and are those most in need of targeted pre-travel health advice.

Of note in 2008, cases of *P. falciparum *malaria, respectively imported from Bahamas [13], and the Dominican Republic [14], and reported to ProMed by the Hamburg site, intensified surveillance by health professionals, but also highlighted differences in malaria chemoprophylaxis recommendations across Europe This is also the case for India [15] (3 cases reported herein). An interesting paper by Calleri et al describes the variability of malaria chemoprophylaxis among "experts" in Europe [16].

**Figure 4:**
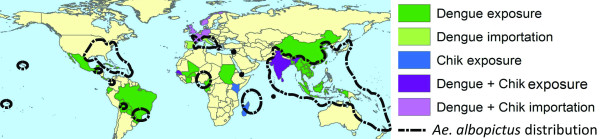
**Countries of acquisition and countries of importation of dengue and chikungunya cases reported by EuroTravNet core sites in 2008 and the overlapping area of habitation of *Aedes albopictus***.

Increased dengue activity was reported throughout endemic areas of the world during 2008, including South Central Asia, notably India, and South America, notably Brazil as compared to 2007 [17]. Several cases were reported from Asia, including cases in travellers to Bali, Indonesia, the Philippines, Thailand, Viet Nam and Myanmar. Five cases were imported into Marseille, France and one case into Brescia, Italy, close to areas inhabited by the mosquito vector *Aedes albopictus*, with the potential threat of onward transmission in Europe [18]. Figure 4 shows a map of the countries of acquisition and countries of importation of dengue cases reported by EuroTravNet core sites and the overlapping area of habitation of *Ae. albopictus *[18].

In addition to mosquito bite prevention for all travellers to endemic areas, it is also essential to stress the potential risk of dengue for MVRA workers in specific circumstances, such as those working in Myanmar as part of the international relief effort in Bogale. This delta region was flooded by the tidal wave which followed Cyclone Nargis in May 2008. After the index case reported in London [19], three more cases were diagnosed in returned travellers from Myanmar by EuroTravNet core sites in 2008.

Chikungunya virus (CHIKV) infection emerged in the field of travel medicine in 2005-2009, since an epidemic spread to the islands throughout the Indian Ocean and to India [20]. More than a thousand cases of imported CHIKV infection in travellers returning from La Reunion (French territory) and other Indian Ocean islands have been reported by France, but also by many other European countries. Recent cases have been diagnosed in travellers returned from tourist destinations where outbreaks re-emerged, such as Thailand [21]. The justified concern in Europe regarding the risk of introduction and transmission of CHIKV was illustrated in 2007 when a single returned traveller was identified as the cause of an outbreak in August 2007 in Italy [4]. At this time there was evidence for indigenous transmission via *Ae. albopictus *mosquitoes that are very active during the summer in this area, and have been shown to be efficient vectors in the transmission of the CHIKV variant responsible for the recent and ongoing outbreak [20]. The presence of *A. albopictus *in areas in Europe makes early detection of imported cases of CHIKV infection essential to prevent emergence of this disease. Of note, 5 of the 12 cases of CHIKV reported here were imported into Marseille, France, less than 150 km from areas where *Ae. albopictus *has been detected.

Chagas disease (CD) is endemic in Central and South America where it affects around 16 to 18 million people. This parasitic infection caused by *Trypanosoma cruzi *has also emerged in the field of travel medicine [22]. Worldwide, Spain is second to the US in having the largest number of immigrants from Latin America. In 2008, a total of 1,607,699 were from *T. cruzi *endemic areas; of these, 239,942 are from Bolivia, the country with the highest prevalence of *T. cruzi *infection. Up to 10% of Bolivian blood donors in Spain had antibodies to *T. cruzi *[23]. This is also illustrated by the majority of cases of CD reported by EuroTravNet in 2008 that were imported from Bolivia to Spain. Given that the infection can be transmitted in non-endemic countries congenitally, by organ donations and blood transfusions, Chagas disease could be regarded as an emerging public health problem in Europe and centres seeing immigrants from Latin America need to be sensitised to the epidemiology of this disease [24].

Tuberculosis is of concern for European public health authorities. In 2006, the EuroTB network reported 88,113 cases of TB in the EU. As reported here, high numbers of cases are of complicated forms, including MDR or XDR TB, and reported amongst vulnerable populations, such as foreign-born VFRs and immigrants. ECDC has published a Framework Action Plan to Fight TB in the EU[25], which provides a roadmap to better control and ultimately eliminate TB in the EU. EuroTravNet sites will continue to play an important role in the surveillance of this disease.

The pre-travel consultation is an opportunity to review a traveller's vaccination history, including both routine schedule vaccines and travel-specific vaccines. In this study, a large number of potentially vaccine-preventable diseases were reported (166 cases). In the cases of diphtheria, the mother had not received pre-travel advice. During the 1990 s, the incidence of diphtheria rose in the newly independent states of the former Soviet Union, due in part to a failure of diphtheria control following the break-up of the Soviet Union. In Eastern Europe (Russian Federation, Ukraine, and other countries of the former Soviet Union, including Latvia) the number of cases has declined since the outbreaks in the 1990 s [26]. Cases of diphtheria may occur in unvaccinated travellers to endemic regions, especially VFRs.

Given the number of cases of Influenza-like illnesses reported to EuroTravNet in 2008, and the experience gained in the H1N1 pandemic, vaccination against influenza should continue to be offered to all travellers at risk of complications from this illness. Healthy individuals travelling to tropical and sub-tropical regions, notably SSA, can also consider vaccination as influenza has been shown to be one of the most frequently occurring vaccine preventable diseases [27]. The issue of the use of appropriate hemisphere influenza vaccine needs to be highlighted as a travel health issue [28]. Travellers should be reminded of the importance of hand hygiene and avoid travel if they have a respiratory illness.

This report also confirms the increasing importance of rickettsioses in ill returned travellers, particularly African tick-bite fever that afflicts travellers to SSA [29] who have often visited safari and game park areas. It also highlights the potential rabid animal-related injury [30], and the importance of including rabies prevention advice and consideration of pre-exposure vaccination during the pre-travel consultation[31]. Finally, the incidence of tick-borne encephalitis (TBE), the most important flavivirus infection of the central nervous system in Europe and Russia [32], is increasing. The risk remains relevant for unvaccinated travellers and illustrates the need for pre-travel advice for travellers within Europe [33].

## Conclusions

As Europe is the worldwide continental leader in international tourism expenditure, the practice of travel medicine, and the framework for guidelines, is of increasing importance. Our study provides a comprehensive analysis of the epidemiology of travel related illness in Europe and opens the door for prospective studies and surveillance of specific diseases of concern within Europe. Aside from gastro-intestinal diseases, febrile systemic illnesses, dermatological conditions and respiratory illnesses, the spectrum of disease in returned travellers is complex. Certain travel destinations are associated with a higher proportionate morbidity for certain diseases. The country of origin of settled immigrants in Europe has a major role to play in disease profiles; individuals presenting with Chagas Disease to EuroTravNet core sites are usually migrants from Bolivia and *P. falciparum *malaria occurs predominantly in migrant families who originate in sub-Saharan Africa and the Indian Ocean Islands.

This study shows that travel within and outside Europe has many implications for health and such data are key to prioritising pre-travel intervention strategies and post-travel decision making.

The spectrum of travel associated morbidity shows that there is a need to dispel the misconception that travel, close to home, in Europe, is without significant health risk. Pre-travel health advice is also clearly indicated for travel within Europe. In 2010, EuroTravNet, commissioned by ECDC, will be working on "*Travel health country information for travel within EU/EFTA"*, in order to provide comprehensive guidance on travel health risks within Europe for travellers and health professionals.

## Abbreviations

(CNS): Central nervous system; (CD): Chagas disease; (CHIKV): Chikungunya virus; (ECDC): European Centre for Disease Prevention & Control; (EU): European Union; (GI): Gastro-intestinal; (GU): Genito-Urinary; (ILIs): Influenza-like illnesses; (MVRAs): Missionary/volunteer/research/aid work; (MDR/XDR TB): Multi-drug resistant/extensively drug resistant tuberculosis; (PPD): Purified protein derivative; (PEP): Post-exposure prophylaxis; (STI): Sexually transmitted infections; (SSA): Sub-Saharan Africa; (TBE): Tick-borne encephalitis; (UK): United Kingdom; (US CDC): United States Centers for Disease Control and Prevention; (VFRs): Visiting friends and relatives; (WHO): World Health Organization.

## Competing interests

The authors declare that they have no competing interests.

## Authors' contributions

VF contributed to the data collection and analysis, and interpretation and first drafted the paper; Ph G, P.S. contributed to the interpretation of the analyses, drafting and reviewing the paper; L.W. made the statistical analysis and reviewed the paper; GDB, EC, MJ, FC, EGK, RLV, PJdV, FvS, and LL contributed to the interpretation of the results and reviewed the paper; P.P. coordinated the work, contributed to study conception and design, the interpretation of the analyses and reviewed the paper. All authors, external and internal, had full access to all of the data (including statistical reports and tables) in the study and can take responsibility for the integrity of the data and the accuracy of the data analysis. All authors read and approved the final manuscript.

## Financial support

EuroTravNet http://www.eurotravnet.eu is the European Centre for Disease Prevention and Control corresponding network for tropical and travel medicine that has been funded through the public tender OJ/2008/07/08-PROC/2008/019. It has been created by grouping the European sites of Geosentinel http://www.geosentinel.org, the Global Surveillance Network of the International Society of Travel Medicine, supported by Cooperative Agreement U50 CI000359 from the US Centers for Disease Control and Prevention.

## Pre-publication history

The pre-publication history for this paper can be accessed here:

http://www.biomedcentral.com/1471-2334/10/330/prepub
